# Conserved and Unique Mitochondrial Target Sequence of TRPV4 Can Independently Regulate Mitochondrial Functions

**DOI:** 10.1002/prot.26772

**Published:** 2024-12-08

**Authors:** Tusar Kanta Acharya, Parnasree Mahapatra, Shamit Kumar, Nishant Kumar Dubey, Srujanika Rajalaxmi, Arijit Ghosh, Ashutosh Kumar, Chandan Goswami

**Affiliations:** ^1^ National Institute of Science Education and Research Bhubaneswar, School of Biological Sciences Khurda Odisha India; ^2^ Homi Bhabha National Institute, Training School Complex Mumbai India

**Keywords:** Ca^2+^‐signaling, channelopathy, ion channel, molecular evolution, oxidative potential

## Abstract

Though mitochondria have their own genome and protein synthesis machineries, the majority of the mitochondrial proteins are actually encoded by the nuclear genome. Most of these mitochondrial proteins are imported into specific compartments of the mitochondria due to their mitochondrial target sequence (MTS). Unlike the nuclear target sequence, the MTS of most of the mitochondrial localized proteins remain poorly understood, mainly due to their variability, heterogeneity, unconventional modes of action, mitochondrial potential‐dependent transport, and other complexities. Recently, we reported that transient receptor potential vanilloid subtype 4 (TRPV4), a thermosensitive cation channel, is physically located at the mitochondria. Here we characterize a small segment (AA 592‐630) located at the TM4‐loop4‐TM5 segment of TRPV4 that acts as a novel MTS. The same region remains highly conserved in all vertebrates and contains a large number of point mutations each of which causes an diverse spectrum of diseases in human. Using confocal and super‐resolution microscopy, we show that this MTS of TRPV4 or its mutants localizes to the mitochondria independently and also induces functional and quantitative changes in the mitochondria. By using conformal microscopy, we could detect the presence of the MTS region within the isolated mitochondria. These findings may be important to understand the complexity of MTS and TRPV4‐induced channelopathies better.

**Abbreviations**
4αPDD: 4α‐phorbol 12,13‐didecanoate (agonist used to activate TRPV4)
GFP: green fluorescent protein (fluorescence protein used for detection of protein localization)
mTP: mitochondrial presequence (scoring used to predict)
MTS: mitochondrial target sequence
NLS: nuclear localization signals
PBS: phosphate‐buffered saline
RFP: red fluorescent protein (fluorescence protein used for detection of protein localization)
ROI: region‐of‐interest (used for image analysis)
ROS: reactive oxygen species (signaling molecules)
TMRM: tetramethylrhodamine, methyl ester (dye used for detecting mitochondrial membrane potential)
TRP: transient receptor potential (group of ion channels)
TRPV4: transient receptor potential vanilloid subtype 4 (a thermosensitive ion channel)

## 1 Introduction

TRPV4 is a thermo‐sensitive, polymodal, nonselective cation channel, belongs to the TRP superfamily, and is involved in various physiological functions. TRPV4‐mediated functions are relevant in neurons, cardiac muscles, bone, immune cells, male gametes, and many other tissues and cells where energy requirements are relatively high [1, 2, 3]. Different point mutations in TRPV4 is linked to diverse pathophysiological conditions [4]. TRPV4 is activated by a number of physical factors such as a hypotonicity, temperature, and chemical factors such as by specific synthetic compound, namely by 4αPDD, and also by several endogenous metabolites. Activation of TRPV4 allows Ca^2+^‐influx within the cell. Surface expression of TRPV4 depends on multiple factors, such as membrane cholesterol, vesicle trafficking, and endosomal recycling [5]. However, specific trafficking of TRPV4 to the cell surface and different subcellular compartments is still poorly understood. The relative presence of TRPV4 in different subcellular organelle‐specific membranes, especially in different conditions, still remains, unknown [6].

Recently, the presence of TRPV4 in the mitochondria of different cellular systems, such as in CHO, Saos‐2, MC3T3‐E1 cells, murine bone marrow‐derived mesenchymal stem cells, bone marrow‐derived macrophages, and T cells, has been reported [7, 8, 9]. Activation of TRPV4 also causes increased mitochondrial Ca^2+^‐levels, both in intact cells and in isolated mitochondria [7, 8, 9]. In a similar context, we observed that TRPV4 interacts with mitochondrial fission‐fusion regulatory factors such as MFN1/MFN2, and modulation of TRPV4 alters the ER‐mito contact points [8]. A small fragment of TRPV4 interacts with Cytochrome C (a mitochondrial protein that exists specially within the inner and outer mitochondrial membrane), more in a metal ion‐dependent/sensitive manners [10]. Based on all these findings, it appears that TRPV4 is involved in mitochondrial functions that are critical in secondary cell lines, primary cells, and stem cells, as well as in male gametes. Nevertheless, the mechanism of translocation of TRPV4, a six‐transmembrane protein, into the mitochondria remains obscure. In this study, we explored if TRPV4 has any sequence that can act as a functional mitochondrial target sequence (MTS). We demonstrate that TRPV4 possesses a novel, unique, and internal MTS (amino acid 592‐630), which is mostly conserved in vertebrates and is sufficient to target to the mitochondria.

## 2 Materials and Methods

### 2.1 In Silico Identification of Novel MTS in TRPV4

For the prediction of the presence of mitochondrial target sequence in TRPV4, WoLF PSORT II (https://psort.hgc.jp/form2.html) software was used. This software predicts the score for the subcellular localization. Another software, iPSORT (https://ipsort.hgc.jp/index.html) is used for the prediction of mitochondrial target sequences in the hTRPV4 full‐length sequence. In a similar manner, the prediction of full‐length hTRPV4 and various hTRPV4 point mutants in to mitochondria has been carried out using Deeplock 2.0 software (https://services.healthtech.dtu.dk/services/DeepLoc‐2.0/).

*Conservation analysis*: To determine whether the mitochondrial targeting signal of hTRPV4 is conserved across species, the hTRPV4 sequence (AA 592‐630) was aligned in Mega 11 software with full‐length TRPV4 from 130 different species (Table S1). R program was used to analyze the conservation of TRPV4 fragment, full‐length TRPV4, and full‐length Histone sequence as described before [11].

*Reagents, cell culture, and transfection*: MitoTracker Red, CMX‐Ros, and TMRM dyes were purchased from Molecular Probes (Invitrogen). HaCaT and Saos‐2 cells were grown in DMEM and McCoy's media. These media were supplemented with FBS (10% v/v), L‐glutamine (2 mM), Penicillin (100 units/mL), and Streptomycin (100 μg/mL). Cells were cultured in a humidity‐controlled incubator maintained at 5% CO_2_ and 37°C. For transient expression, Lipofectamine 3000 Plus reagent (Invitrogen) was used as per the instruction provided by the manufacturer. Live cell imaging or immunocytochemistry was performed after 24–36 h of transfection.

*Constructs and vector used*: In many experiments, the TRPV4‐GFP construct (cloned in the pEGFPN3 vector, kindly offered by Dr. J.B. Hahn) was used [12]. The generation of different point mutations was described before [9]. The point mutations were created by using site‐directed mutagenesis by using kit from Agilent Technologies. The mitoDsRed construct was purchased from Addgene.

### 2.2 Live Cell Imaging and Confocal Microscopy

For the live cell imaging, cells were grown on 25‐mm glass cover slips and subsequently transfected by using Lipofectamine 3000 (ThermoFisher). Two days after seeding or transfection, cells were labeled with either Tetramethylrhodamine, methyl ester (TMRM, 50 nM) or MitoTracker Red (0.5 μM) for 20 min as per the requirement. The unbound dyes were washed by Phosphate‐Buffered Saline (PBS) and subsequently the cells were imaged by a confocal microscope (Olympus FV3000) using the 63× objective. Live cell images were acquired in a temperature‐ and humidity‐controlled live cell imaging chamber (Oko lab). Images were analyzed and processed by ImageJ.

### 2.3 Super Resolution Imaging and Image Processing

For super resolution microscopy, Saos‐2 cells were seeded to the different coverslips, and TRPV4‐WT‐MTS‐GFP, TRPV4‐L618P‐MTS‐GFP, and TRPV4‐L596P‐MTS‐GFP were expressed by transient over expression. After 12 h of transfection, cells are treated with MitoTracker Red and fixed by using 4% paraformaldehyde and kept at room temperature. Coverslips are mounted to the slides by Fluoromount‐G, and lattice SIM super resolution images are acquired by using a Zeiss elyra 7 microscope, and images were processed by ZEN Black software.

### 2.4 Mitochondria Isolation and Imaging

TRPV4‐WT‐MTS‐GFP or TRPV4‐L618P‐MTS‐GFP were transiently over expressed in Saos‐2 cells. Subsequently, mitochondria were isolated by using the QProteome Mitochondria isolation kit (Qiagen, Germany) by following the standard protocol provided by the manufacturer. The isolated mitochondria are incubated with the MitoTracker Red dye (1 μm) for 30 min at 37°C. Live mitochondrial imaging is performed by using a confocal microscope, and images are taken from multiple view fields. 75 × 75 pixel from 4× magnified images were cropped for image representation.

### 2.5 Quantification of Mitochondrial Morphological Parameters

For the quantification of the parameters related to mitochondrial morphology, mitochondrial morphology plugin (https://imagejdocu.tudor.lu/plugin/morphology/mitochondrial_morphology_macro_plug‐in/start) was used as described before [9, 13]. Different parameters related to mitochondrial morphology, such as average area, perimeter, area/perimeter, mito‐content, mitochondrial number, average circularity, and solidity were quantified. For mitochondrial translocation study, > 1000 individual mitochondrial Region‐of‐Interests (ROI) were quantified.

## 3 Results

### 3.1 Identification of the MTS Sequence of TRPV4

To identify the presence of possible mitochondrial target sequence (MTS) within TRPV4, the full‐length hTRPV4 and its systemic deletion sequence stretches were analyzed with several prediction programs to determine their best possible subcellular localization and the target sequence. The full‐length TRPV4 sequence has no significant score that can be employed as an MTS. Thereafter, the full‐length hTRPV4 and its systemic deleted sequences were then analyzed using the WoLF PSORT II (https://psort.hgc.jp/form2.html) software to evaluate the presence of any possible MTS sequence. Each sequence stretches received a separate score for probable localization in organelles (Table S2). This software examined a total of 19 distinct deletion sequences for mitochondrial target sequences. The prediction results indicate that amino acid 592‐630 of TRPV4 has the highest score for possible mitochondrial targeting when compared to other stretches. Similarly, using the iPSORT prediction software, it was observed that full‐length hTRPV4 apparently lacks conventional mitochondrial signal and a mitochondrial‐targeting peptide, yet the amino acid sequence 592‐630 fragment is recognized as a potential mitochondrial‐targeting peptide (Table S3).

Furthermore, we used Deeplock 2.0 software to investigate how the presence of numerous naturally occurring point mutants in the region of predicted mitochondrial‐target sequences affects the score of mitochondrial localization frequency. This website prediction for mitochondrial presequence (mTP) score is represented (Table S4). Greater the mTP score, higher the prediction for its localization into the mitochondria. The Deeplock 2.0 score indicates that the point mutants, such as L596P, R616Q, F617L, L618P, V620I, TRPV4‐MTS (592‐630 aa), and TRPV4‐C terminus (718‐871 aa), have a higher mTP score and suggest a higher translocation probability to the mitochondria. But the full‐length TRPV4 (1‐871aa), TRPV4‐TM region (466‐711aa), and TRPV4‐N terminal (1‐465aa) show a lesser mTP score. However, our previous results indicated that full‐length TRPV4 is also present in the mitochondria [2, 9].

### 3.2 Analysis of Conservation of Predicted MTS Sequence in all Vertebrates

**FIGURE 1:**
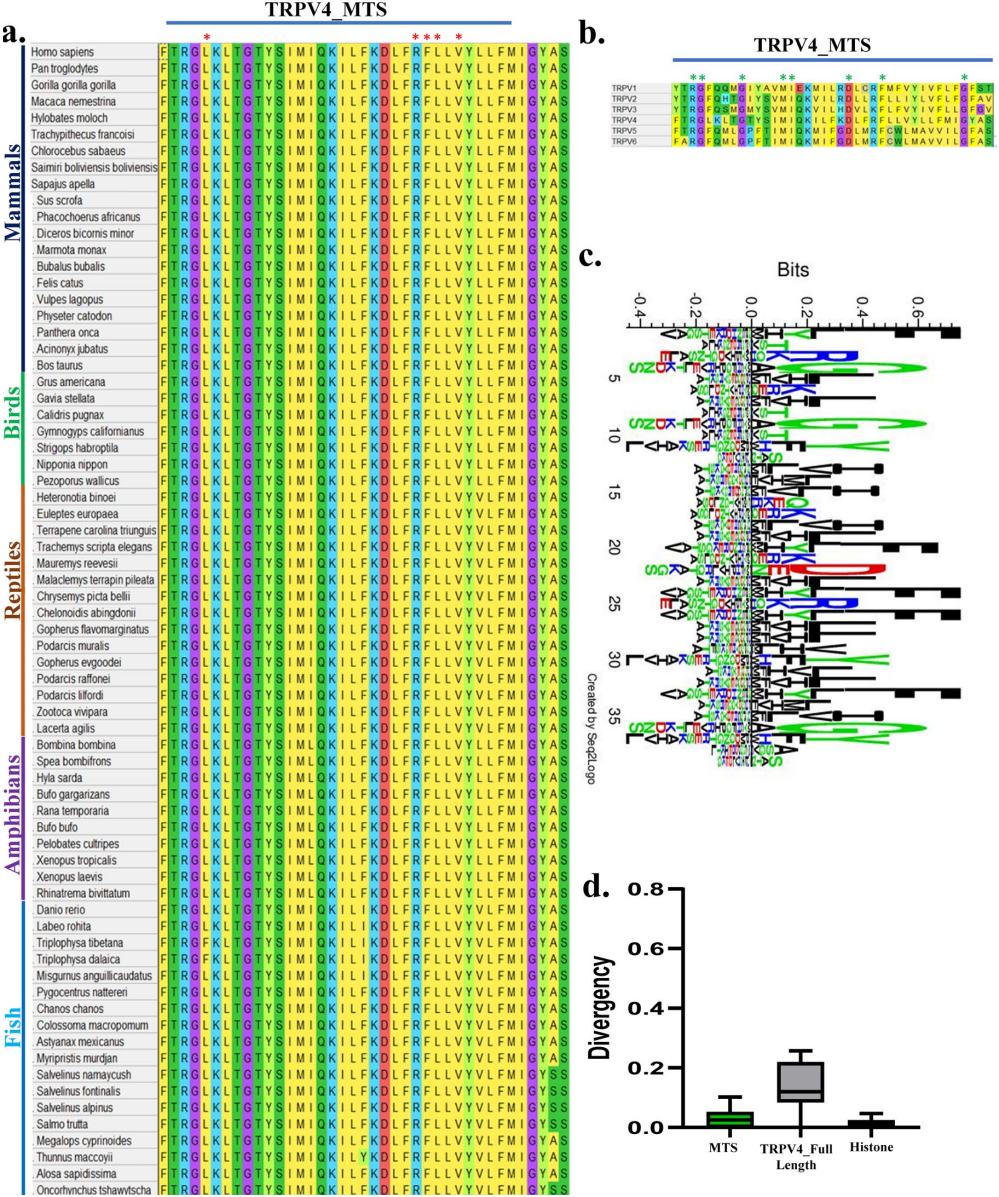
The MTS sequence of TRPV4 remains conserved in all vertebrates. (a). The full‐length protein sequence of TRPV4 from 131 species was aligned in Mega 11 software, and amino acid stretch 592‐630 from 70 species only (representing all five vertebrate classes) is shown here. The sequence alignment indicates that the TRPV4‐MTS sequence is conserved throughout the vertebrate evolution. The red asterisks indicate pathogenic mutations identified in the human population. For more details, see Figure S1. (b). The MTS sequence of TRPV4 is compared with other TRPV members. The green asterisks indicate common residues in all TRPV members. (c). Seqlogo of the TRPV4‐MTS region among 131 vertebrate species are shown. (d). Conservation analysis (box‐plot of divergence from 131 vertebrate species) of the MTS sequence (592‐630) in comparison to full‐length TRPV4 (871 aa) is shown. In this analysis, the higher the value, the lesser the conservation and *vice versa*. The MTS region is more conserved that the full‐length TRPV4.

In silico prediction indicates that amino acid 592‐630 of TRPV4 (FTRGLKLTGTYSIMIQKILFKDLFRFLLVYLLFMIGYAS) has the maximum probability for mitochondrial localization (Figure 1a). We attempted to determine if the MTS of hTRPV4 is conserved across all species. For that purpose, we have aligned the full‐length TRPV4 protein sequence in Mega 11 software from 131 species. Alignment data shows that amino acid 592‐630 sequence stretch is highly conserved throughout the vertebrate evolution. However, in some fishes, alignment was substituted with similar amino acids (Figure 1a). Some of the residues of this sequence are also conserved in other TRPV channels too (Figure 1b). We analyzed the conservation of amino acid 592‐630 sequence stretch with respect to full‐length TRPV4 (871 aa), as done before [11] (Figure 1c). The box‐plot analysis of divergence from more than 130 species was performed. The data suggests that the TRPV4‐(592‐630) region is more conserved than that of the full‐length TRPV4 sequence, suggesting a stronger selection pressure on this segment during the vertebrate evolution (Figure 1d).

### 3.3 TRPV4 Has a Novel MTS Sequence Which Localizes to the Mitochondria

**FIGURE 2:**
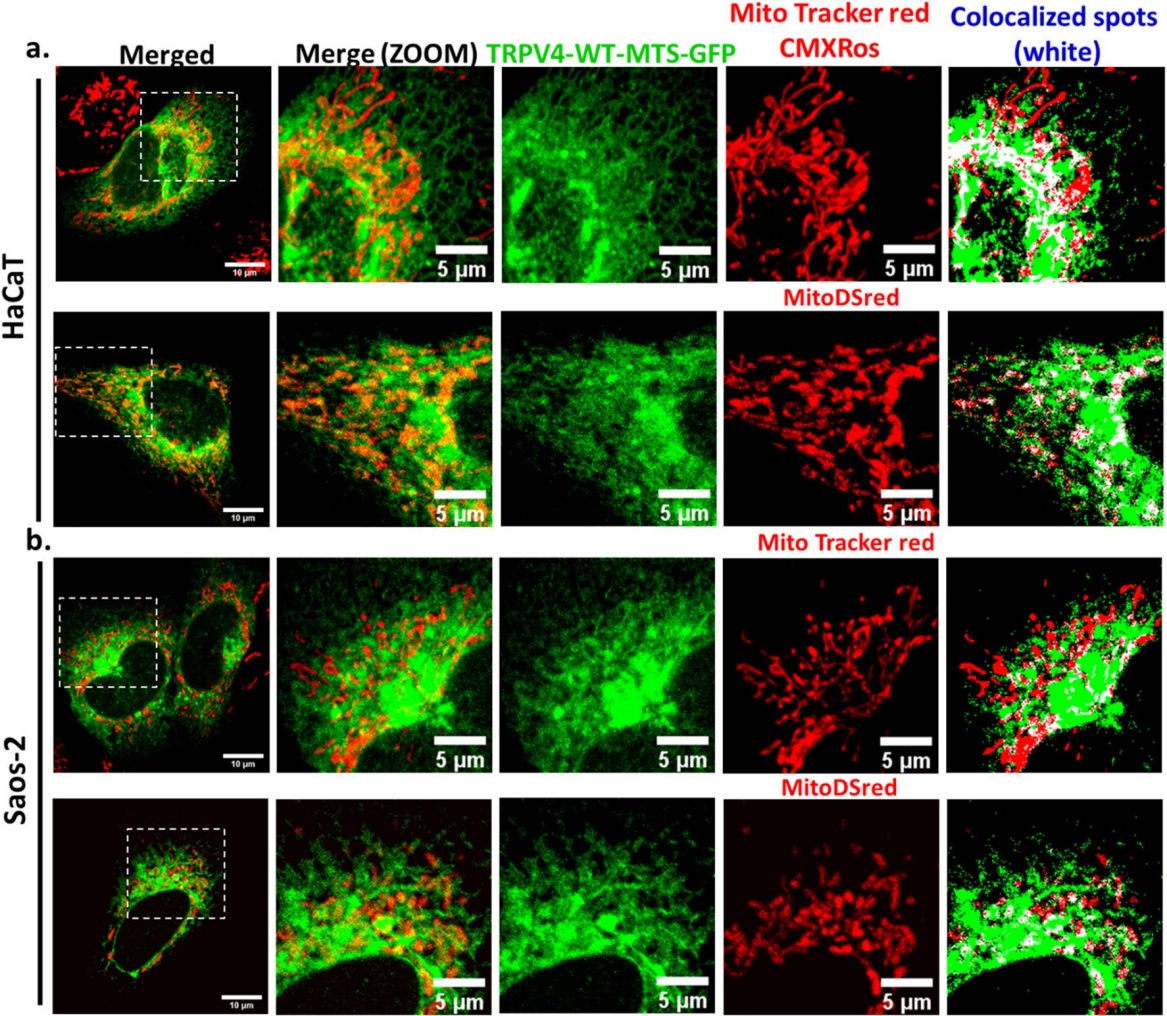
The MTS sequence of TRPV4 is able to localize to the mitochondria. (a, b). TRPV4‐(592‐630)‐GFP was transiently expressed in HaCaT (a) or Saos‐2 (b) cells along with mitoDsRed or stained with MitoTracker Red. The colocalized pixels are shown in white.

To validate if the in silico predicted sequence stretch can truly localize to the mitochondria, we cloned this sequence (AA number 592‐630) into the pEGFP‐N3 vector [termed as TRPV4‐(592‐630)‐GFP]. We expressed this segment in HaCaT and Saos‐2 cells along with the mitoDsRed or MitoTracker Red CMX‐Ros (as mitochondrial markers) and carried out colocalization experiments. These experiments suggest that TRPV4‐(592‐630)‐GFP colocalizes with mitoDsRed or MitoTracker Red, and this fragment is present in mitochondria. We therefore named this amino acid stretch (592‐630) as TRPV4‐MTS (Figure 2a,b).

### 3.4 Different TRPV4‐MTS Mutants Localize in the Mitochondria in HaCaT Cells

Previous reports suggest that there are different point mutations that are located in the MTS region of TRPV4, and each of these point mutations is known to induce different channelopathies. Among all these, few mutants produce gain‐of‐function‐type while most of these mutations lead to several pathophysiological disorders [14]. Since all these mutations are located within this MTS sequence, we investigated these naturally occurring mutants with respect to mitochondrial targeting signal as well as their physiological functions. In this context, five different point mutations (namely, L596P, R616Q, F617L, L618P, and V620I) were expressed in HaCaT cells by cloning into the pE‐GFP‐N3 vector along with the mitoDsRed. This allowed us to understand their mitochondrial localization and mitochondrial integrity.

**FIGURE 3:**
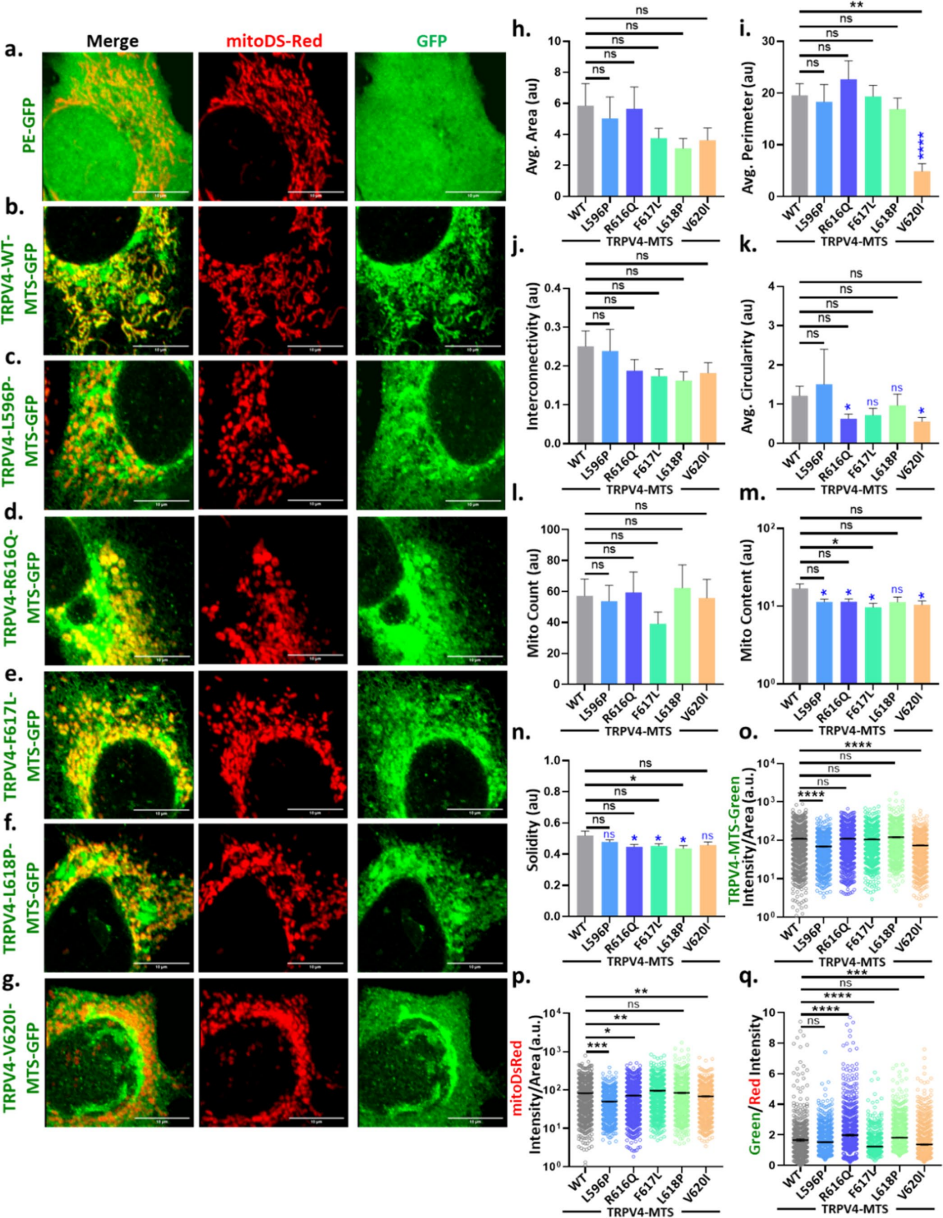
TRPV4‐MTS mutants predominantly colocalize with mitochondria. (a–g). TRPV4‐WT‐MTS and different TRPV4‐MTS mutants (TRPV4‐L596P‐MTS‐GFP, TRPV4‐R616Q‐MTS‐GFP, TRPV4‐F617L‐MTS‐GFP, TRPV4‐L618P‐MTS‐GFP, and TRPV4‐V620I‐MTS‐GFP) along with mitoDsRed (as a mitochondrial marker) were expressed in HaCaT cells by transient transfection. The mitochondrial shape becomes atypical in the cells expressing these mutants as compared to cells expressing TRPV4‐WT or pEGFP. (h–n). Different mitochondrial parameters, as mitochondrial area (h), perimeter (i), interconnectivity (j), circularity (k), mito‐count (l), mito‐content (m), and solidity (n) were quantified. (o–q). Enrichment of TRPV4‐MTS in mitochondria was assessed by quantifying the TRPV4‐GFP fluorescence intensity over the mitoDsRed fluorescence intensity (> 1000 mitochondrial particles from > 10 cells were quantified in each condition). One‐way ANOVA, ns = nonsignificant, *p < 0.05; **p < 0.01; ***p < 0.001; ****p < 0.0001.

The cells expressing these MTS mutants show colocalization with mitoDsRed and have altered mitochondrial shape and size. In cases of these mutants, the mitochondria become mostly round‐shaped instead of their reticulate structure as observed in TRPV4‐WT‐MTS cells (Figure 3a–g).

Next, we quantified alteration in different mitochondrial morphological parameters such as mitochondrial area, perimeter, interconnectivity, circularity, number, content, and solidity in cells expressing MTS of WT and mutants in HaCaT cells. For all these comparative purposes, TRPV4‐MTS‐GFP is used as a reference rather than the GFP‐only. This is appropriate as GFP is smaller than MTS‐GFP, the expression level of GFP is much higher than any of the constructs, and GFP appears to be more diffusely distributed all over the cells nonspecifically.

We observed that there is differential alteration of mitochondrial morphology in different mutations (Figure 3h–n). Furthermore, we measured the extent of translocation of TRPV4‐MTS (WT as well as different mutants) into the mitochondria (Figure 3o–q). For this purpose, we quantified the intensity of individual mitochondria (mitoDsRed) and TRPV4 WT and mutant intensity. The ratio of TRPV4‐MTS (as GFP‐tagged protein)/mitoDsRed indicates that TRPV4‐R616Q‐MTS, TRPV4‐F617L‐MTS, and TRPV4‐V620I‐MTS mutants have higher translocation efficiency as compared to TRPV4‐WT‐MTS (Figure 3o).

**FIGURE 4:**
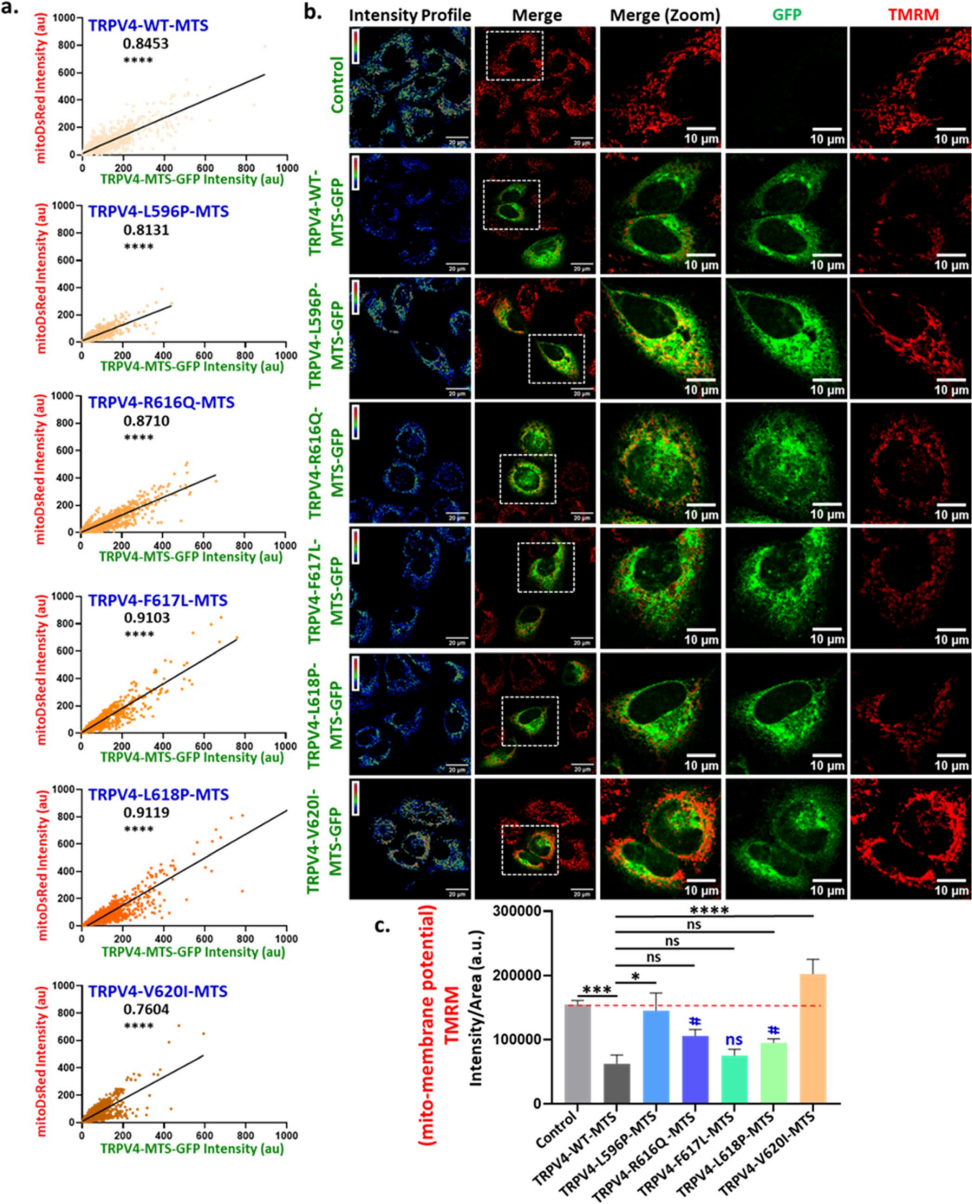
Different TRPV4‐MTS mutants affect the extent of mitochondrial translocation and regulate mitochondrial membrane potential. (a). The graphs represent the correlation (*r*) of various TRPV4‐MTS mutants with mitoDsRed. ****p < 0.0001. Some of the mutants have better mitochondrial localization than TRPV4‐WT. (b). Confocal images of HaCaT cells expressing TRPV4‐WT‐MTS or different naturally occurring mutants located in the MTS region as GFP‐tagged protein (green) are shown. Cells were subsequently labeled with TMRM (red) for detection of change in mitochondrial membrane potential. (c). Quantification of TMRM‐intensity in different cell population depicts that expression of MTS‐WT or mutants affects mitochondrial membrane potential differently. *n* > 25 cells in each condition. One‐way ANOVA, ns = non‐significant, *p < 0.05; ***p < 0.001; ****p < 0.0001.

We also evaluated the extent of different mutants of TRPV4‐MTS for translocation to mitochondria by calculating the correlation values (*r*) of TPRV4‐MTS with mitoDsRed intensity (Figure 4a). The analysis reveals that R616Q, F617L, and L618P TRPV4‐MTS mutants have higher *r* values as compared to TRPV4‐WT‐MTS. Overall, the data suggests that different TRPV4‐MTS mutants affect both ability and extent of colocalization with mitochondria.

### 3.5 TRPV4‐MTS Mutants Regulate Mitochondrial Membrane Potential

Next, we explored the impact of expression of TRPV4‐WT‐MTS and mutants on mitochondrial health. To address that, we measured mitochondrial membrane potential by using TMRM dye (Figure 4b). By quantifying the change in TMRM intensity, we observed that expression of different TRPV4‐MTS mutants alters the mitochondrial membrane potential differently. Most of the tested mutants show a higher TMRM fluorescence level as compared to cells expressing TRPV4‐WT (Figure 4c).

Taken together, the data identify a small segment of TRPV4 as a novel mitochondrial target sequence that also has a large number of point mutations causing diseases.

### 3.6 TRPV4‐MTS Is Located Both at Surface and Within Mitochondria

**FIGURE 5:**
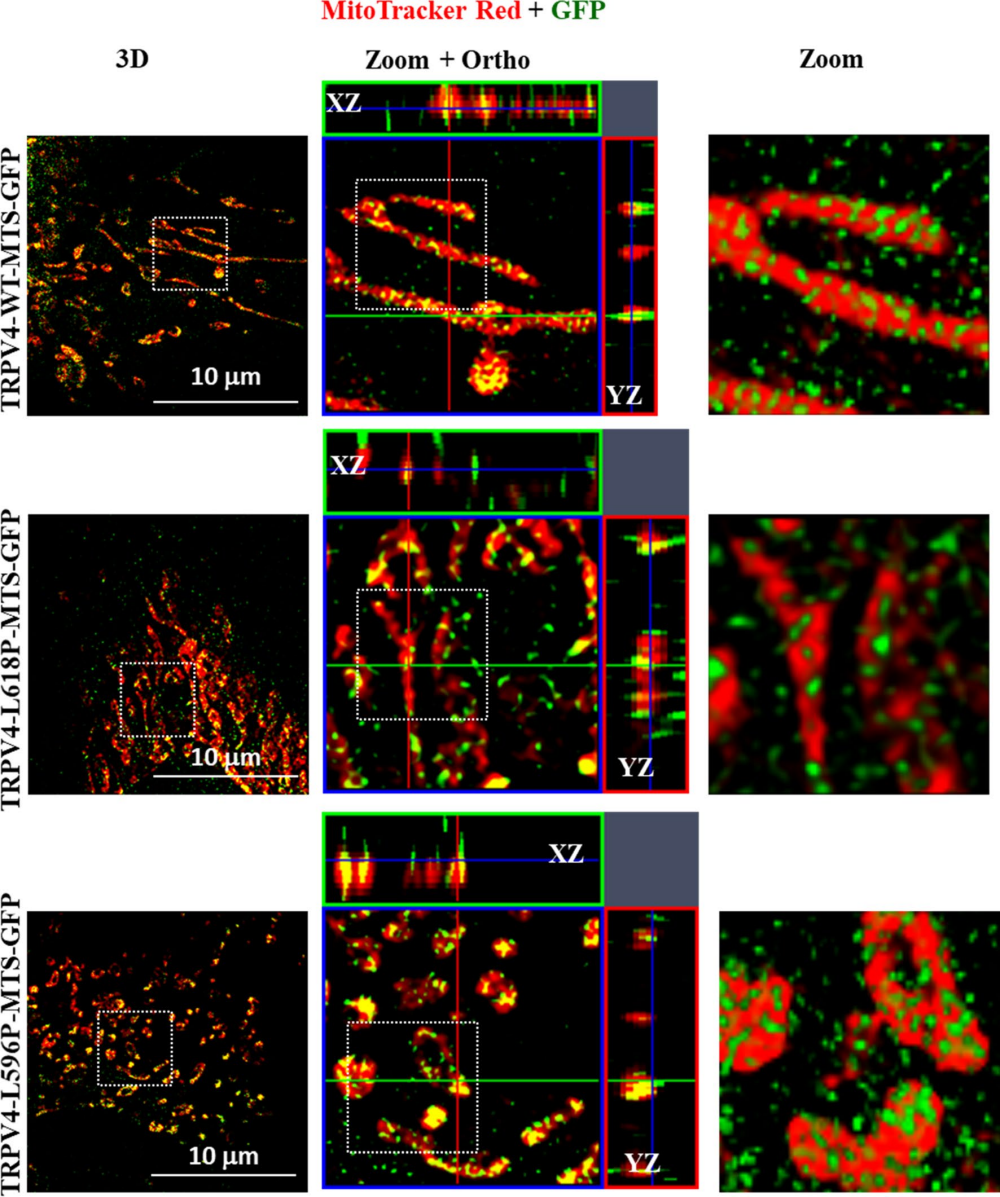
TRPV4‐WT‐MTS‐GFP as well as TRPV4‐L596P‐MTS‐GFP and TRPV4‐L618P‐MTS‐GFP is detected within and on the surface of mitochondria. Shown are the 3D super resolution images (left panel) of Saos‐2 cells transiently expressing TRPV4‐WT‐MTS‐GFP, or TRPV4‐L596P‐MTS‐GFP, or TRPV4‐L618P‐MTS‐GFP and labeled with MitoTracker Red, fixed, and images were acquired by the SIM2 method. Much enlarged section with XZ and YZ orthogonal projection (middle panel) or even further enlarged area are shown (right most panel). MTS‐specific GFP signal is observed both within as well as on the surface of mitochondria.

In order to visualize the presence of TRPV4‐WT‐MTS‐GFP or a mutant form of the same on or in the mitochondria, we expressed TRPV4‐WT‐MTS‐GFP as well as TRPV4‐L596P‐MTS‐GFP and TRPV4‐L618P‐MTS‐GFP in Saos‐2 cells and performed the super resolution imaging. We could detect the GFP signal from TRPV4‐WT‐MTS‐GFP as well as TRPV4‐L596P‐MTS‐GFP and TRPV4‐L618P‐MTS‐GFP on as well as within the mitochondrial structures (Figure 5).

### 3.7 Both WT and Mutant TRPV4‐MTS Is Detected in Isolated Mitochondria

**FIGURE 6:**
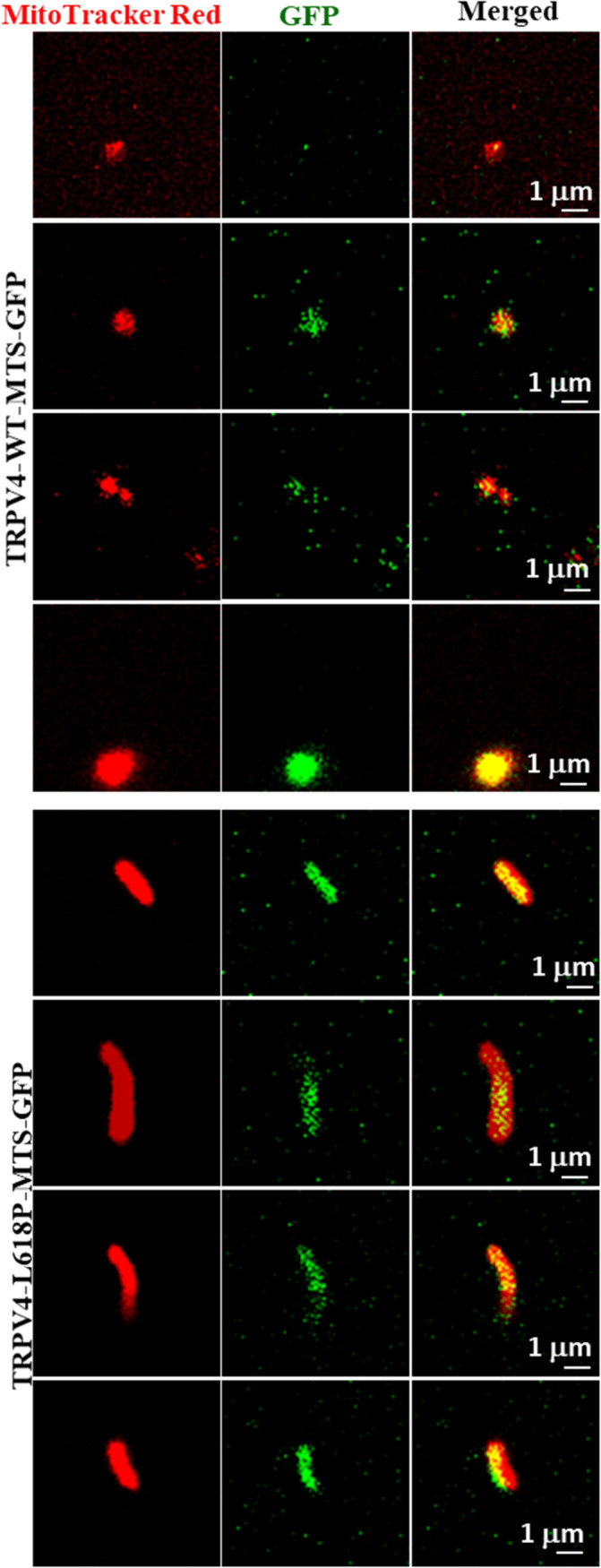
TRPV4‐WT‐MTS‐GFP as well as TRPV4‐L618P‐MTS‐GFP is detected in the isolated mitochondria. Shown are the confocal images of mitochondria isolated from Saos‐2 cells transiently expressing TRPV4‐WT‐MTS‐GFP or TRPV4‐L618P‐MTS‐GFP and further labeled with MitoTracker Red. Images were acquired by a FV‐3000 (Olympus) confocal microscope. Variable levels of MTS‐specific GFP‐signal are observed within the isolated mitochondria, suggesting a differential level of presence of TRPV4‐WT‐MTS‐GFP as well as TRPV4‐L618P‐MTS‐GFP in the mitochondria.

To confirm that TRPV4‐WT‐MTS‐GFP or its mutants are indeed present within the mitochondria, we expressed TRPV4‐WT‐MTS‐GFP, as well as TRPV4‐L618P‐MTS‐GFP transiently in Saos‐2 cells, labeled the cells with MitoTracker Red, and isolated the mitochondria followed by confocal imaging (Figure 6). In both cases, we could detect the GFP fluorescence (though at variable extent and not in all) in the mitochondria isolated from the cells, confirming the presence of TRPV4‐WT‐MTS‐GFP as well as TRPV4‐L618P‐MTS‐GFP in the mitochondria.

## 4 Discussion

Though mitochondria has its own genome, it codes only 37 gene products, out of which 2 code for rRNAs, 22 code for tRNAs, and 13 genes are translated to proteins [15]. Thus, all other proteins present in the mitochondria are encoded by the nuclear genome and subsequently incorporated into the mitochondria. Mammalian mitochondria is expected to harbor more than 1500 proteins with different structures, function, and regulation [16]. The molecular routes and the protein complexes (such as TIM‐ and TOM‐complexes) involved in the protein transport to the mitochondria are relatively well‐established. Specific proteins are translocated to the mitochondria due to the presence of conventional MTS. Classically, the majority of the nucleus‐encoded mitochondrial proteins contain N‐terminal MTS, or presequence [17]. So far several mitochondrial proteins have been identified that has clear mitochondrial target sequences at their N‐terminal regions. Often, the translocation of the proteins with presequence across the inner membrane is regulated by the mitochondrial membrane potential, that is, by the electrochemical force across this membrane (ΔΨ) acting on the positive charges of the presequence [18]. The positively charged residue of MTS formed amphipathic α‐helical structure and has the ability to recognize translocation machinery present in the mitochondria. However, there are proteins where MTS does not form a perfect amphipathic α‐helical structure, yet enters to the mitochondria [19]. Though most of the mitochondrial proteins have MTS located at their N‐terminus, still many mitochondrial proteins differ from classical convention, where MTS‐stretch actually lies either at the C‐terminal region or at the middle of the protein (known as internal targeting signal) [20, 21, 22, 23, 24]. For example, import of mitochondrial β‐barrel proteins is due to the unique MTS that have β‐hairpin structure [25]. Besides having an MTS‐like segment, certain proteins like Bcl‐2 [26] and heme lyase [20] have an internal targeting sequence that allow these proteins to the mitochondrial membrane. There are also examples of nuclear proteins (such as human apurinic/apyrimidinic endonuclease 1) that are targeted to mitochondria when their Nuclear Localization Signal (NLS) region becomes nonfunctional or masked by some other mechanisms [24]. In spite of all these findings, the mitochondrial target sequence of all the mitochondrial proteins is unclear and still poorly understood.

**FIGURE 7:**
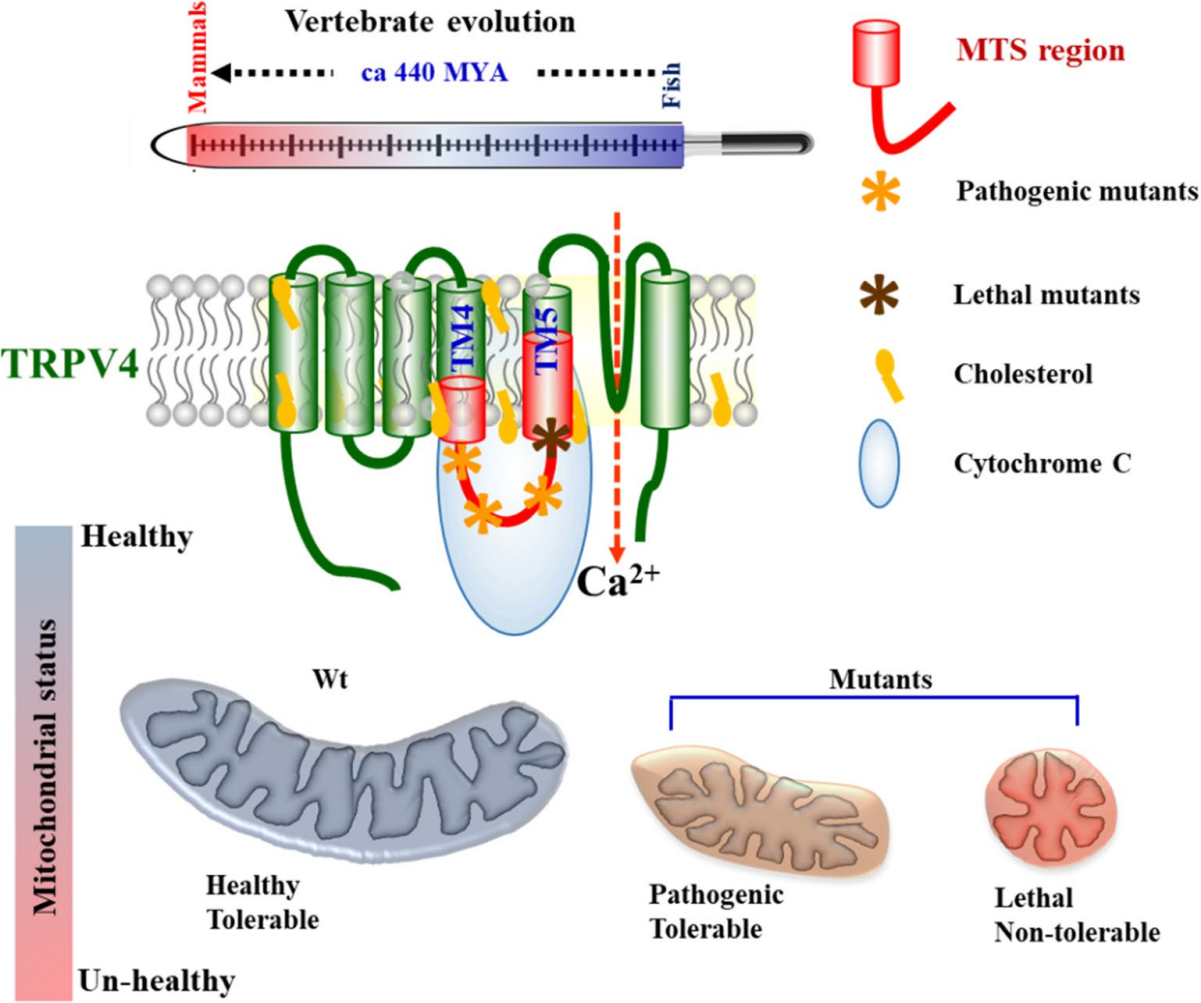
Schematic model depicting the presence of MTS in TRPV4. TRPV4 has an internal sequence at the TM4‐loop4‐TM5 segment, which acts as a novel mitochondrial target sequence. The same segment remains highly conserved in all vertebrates and contains several disease‐causing point mutations with different levels of lethality. This differential pathophysiology is mostly due to their differential effect on a subpopulation of mitochondria.

In this work, we demonstrate that TRPV4 contains a short sequence stretch that can act as a potential and novel MTS (Figure 7). Notably, this MTS region remains highly conserved in all vertebrates and is more conserved than the full‐length TRPV4, suggesting more selection pressure on this region. Upon BLAST search, we could not find any matching with this sequence to any other proteins (other than TRPV4 or a few other distant TRP channels), suggesting that this stretch is unique in its sequence (data not shown). This region also contains several positively charged residues and has the possibility to form an amphipathic helix in certain conditions. Moreover, the same region is known to interact with Cytochrome C as well as different compounds such as mevalonate, cholesterol, progesterone, and testosterone, that is, few metabolites that represent sterol and steroid biosynthesis pathways [10, 27, 28]. Notably, both mitochondrial functions and ER‐mitochondrial contact points are critical for steroid synthesis.

Though we demonstrate that amino acid stretch 592‐630 is capable of driving fusion proteins (such as GFP or RFP) to the mitochondria and several mutations alter such functions qualitatively and quantitatively, the exact molecular mechanism remains unknown. We also want to highlight that when we analyzed the TRPV4 sequence with MTS‐deleted sequence using the same prediction method, we detected no mitochondrial target sequence, and the scores were similar to the full‐length TRPV4 sequence (data not shown). Our experimental result demonstrated that the anticipated MTS sequence is critical for the mitochondrial localization of TRPV4. When expressed as a GFP‐tagged protein, the MTS region is detected in as well as on the surface of the mitochondria, as observed by confocal as well as by super resolution imaging. Notably, TRPC3, another TRP channel, is known to be present in the mitochondria and the exact MTS of TRPC3 also remains unknown [29]. However, analysis of full‐length TRPC3 sequence in various predicting software fails to predict a decent score for the mitochondrial target sequence (except the TRPC3‐S3‐L‐S4 sequence, which shows 30.4% compared to the full‐length 4.3% by PSORT II prediction software) and requires further and detailed investigation. Notably, recently an isoform of TRPV1 has been reported that has a MTS in its N‐terminus [30]. The MTS region of TRPV4 identified here also serves as a “hot‐spot” for several disease‐causing mutations [4]. Previously, we noted that TRPV4 localizes in a subset of mitochondria when expressed ectopically [9]. Regulation of structure and function of a fraction of total mitochondria by TRPV4 also accords well with the differential penetration of different TRPV4 mutations. The phenotypes of all these TRPV4 mutations vary from severe neonatal lethality to mild impact, and thus our finding can be useful to understand the TRPV4‐mutation‐induced channelopathies better.

Though we identified amino acid 592‐630 as a unique mitochondrial target sequence and characterized the same fragment, there are still several intriguing questions that remain unaddressed. For example, retaining a MTS region in the middle of the 6‐TM protein during vertebrate evolution is puzzling. It is not clear if the MTS sequence is processed further within the mitochondria by some proteases during mitochondrial translocation and/or this MTS region has some complex evolutionary background.

Never‐the‐less, these findings may have broad importance in understanding mitochondria‐induced diseases, such as neurodegeneration, obesity, skeletal dysfunctions and possible future therapeutics [31, 32, 33]. Our findings strongly suggest TRPV4 as a key molecular candidate to understand mitochondrial diseases.

## Author Contributions

**Tusar Kanta Acharya:** methodology, software, data curation, writing – original draft, project administration, validation, formal analysis, investigation. **Parnasree Mahapatra:** writing – original draft, methodology, software, data curation, investigation, formal analysis, validation. **Shamit Kumar:** methodology, investigation, validation, formal analysis, writing – original draft, data curation. **Nishant Kumar Dubey:** methodology, software, writing – original draft, validation, investigation. **Srujanika Rajalaxmi:** methodology, formal analysis, validation, software. **Arijit Ghosh:** software, methodology, writing – original draft. **Ashutosh Kumar:** conceptualization, methodology, investigation, visualization, writing – original draft. **Chandan Goswami:** conceptualization, methodology, writing – review and editing, writing – original draft, project administration, resources, supervision, visualization, funding acquisition, investigation, validation.

## Conflicts of Interest

The authors declare no conflicts of interest.

### Peer Review

The peer review history for this article is available at https://www.webofscience.com/api/gateway/wos/peer‐review/10.1002/prot.26772.

## Supporting information


Data S1.

## Data Availability

The data that support the findings of this study can be provided by the authors upon reasonable request.
