# Supplementary material for: Conserved and Unique Mitochondrial Target Sequence of TRPV4 Can Independently Regulate Mitochondrial Functions
Source: Proteins. 2024 Dec 8;93(4):908–19. doi: 10.1002/prot.26772 (PMC11878201; doi:10.1002/prot.26772)
Supplement: Supplementary file 1 — Data S1. [file PROT-93-908-s001.pdf]

Supplementary information

Conserved and unique mitochondrial target sequence of TRPV4 can independently regulate mitochondrial functions

Running title: Conserved mitochondrial target sequence of TRPV4

Tusar Kanta Acharya[1,2,#], Parnasree Mahapatra[1,2,#], Shamit Kumar[1,2,#], Nishant Dubey[1,2,#], Srujanika Rajalaxmi[1,2], Arijit Ghosh[1,2], Ashutosh Kumar[1,2], Chandan Goswami[1,2*]

1. National Institute of Science Education and Research Bhubaneswar, School of Biological Sciences, P.O. Jatni, Khurda 752050, Odisha, India

2. Homi Bhabha National Institute, Training School Complex, Anushakti Nagar, Mumbai 400094, India

# Equal contribution,

* Correspondence: chandan@niser.ac.in

Supplementary table 1 (List of all 131 species selected for analysis of TRPV4)

NP_067638.3	- *Homo sapiens*	Mammal
XP_016779640.1	- *Pan troglodytes*	Mammal
XP_018894818.3	- *Gorilla gorilla gorilla*	Mammal
XP_011759392.1	- *Macaca nemestrina*	Mammal
XP_058281387.1	- *Hylobates moloch*	Mammal
XP_033076571.1	- *Trachypithecus francoisi*	Mammal
XP_008002838.1	- *Chlorocebus sabaeus*	Mammal
XP_039328239.1	- *Saimiri boliviensis boliviensis*	Mammal
XP_032157053.1	- *Sapajus apella*	Mammal
XP_029778041.1	- *Suricata suricatta*	Mammal
XP_032253895.1	- *Phoca vitulina*	Mammal
XP_055179051.1	- *Nyctereutes procyonoides*	Mammal
XP_032954449.1	- *Rhinolophus ferrumequinum*	Mammal
XP_039103792.1	- *Hyaena hyaena*	Mammal
XP_035941826.1	- *Halichoerus grypus*	Mammal
XP_045647427.1	- *Ursus americanus*	Mammal
XP_036730044.1	- *Balaenoptera musculus*	Mammal
XP_055260733.1	- *Moschus berezovskii*	Mammal
XP_013838403.1	- *Sus scrofa*	Mammal
XP_047617350.1	- *Phacochoerus africanus*	Mammal
XP_058387596.1	- *Diceros bicornis minor*	Mammal
KAF7472626.1	- *Marmota monax*	Mammal
XP_006052982.1	- *Bubalus bubalis*	Mammal
XP_023097285.2	- *Felis catus*	Mammal
XP_041585426.1	- *Vulpes lagopus*	Mammal
XP_028336047.1	- *Physeter catodon*	Mammal
XP_060468505.1	- *Panthera onca*	Mammal
XP_026899867.1	- *Acinonyx jubatus*	Mammal
NP_001179314.1	- *Bos taurus*	Mammal
XP_057382733.1	- *Balaenoptera acutorostrata*	Mammal
XP_005340170.1	- *Ictidomys tridecemlineatus*	Mammal
XP_040106847.1	- *Oryx dammah*	Mammal
XP_027972408.1	- *Eumetopias jubatus*	Mammal
XP_025745161.1	- *Callorhinus ursinus*	Mammal
XP_004429938.1	- *Ceratotherium simum simum*	Mammal
XP_047684657.1	- *Prionailurus viverrinus*	Mammal
XP_047554089.1	- *Lutra lutra*	Mammal
XP_027720215.1	- *Vombatus ursinus*	Mammal
XP_036596700.1	- *Trichosurus vulpecula*	Mammal

XP_042817972.1	- *Panthera tigris*	Mammal
XP_037673140.1	- *Choloepus didactylus*	Mammal
XP_051828966.1	- *Antechinus flavipes*	Mammal
XP_042766185.1	- *Panthera leo*	Mammal
XP_003477960.1	- *Cavia porcellus*	Mammal
XP_031804647.1	- *Sarcophilus harrisii*	Mammal
VFV38569.1	- *Lynx pardinus*	Mammal
XP_030148103.1	- *Lynx canadensis*	Mammal
XP_056677571.1	- *Monodelphis domestica*	Mammal
XP_032703293.1	- *Lontra canadensis*	Mammal
XP_045882061.1	- *Meles meles*	Mammal
XP_044100015.1	- *Neogale vison*	Mammal
XP_019613090.1	- *Rhinolophus sinicus*	Mammal
XP_043834665.1	- *Dromiciops gliroides*	Mammal
XP_057162046.1	- *Ursus arctos*	Mammal
XP_006204825.1	- *Vicugna pacos*	Mammal
XP_017397200.1	- *Cebus imitator*	Mammal
NP_001120787.1	- *Canis lupus familiaris*	Mammal
XP_057602488.1	- *Hippopotamus amphibius kiboko*	Mammal
XP_045390545.1	- *Lemur catta*	Mammal
XP_031298753.1	- *Camelus dromedarius*	Mammal
XP_054700856.1	- *Grus americana*	Bird
XP_059683695.1	- *Gavia stellata*	Bird
XP_014795509.1	- *Calidris pugnax*	Bird
XP_050762928.1	- *Gymnogyps californianus*	Birds
XP_030356954.1	- *Strigops habroptila*	Bird
XP_009470591.1	- *Nipponia nippon*	Bird
XP_057269056.1	- *Pezoporus wallicus*	Bird
XP_053136317.1	- *Hemicordylus capensis*	Reptile
NP_001304074.1	- *Alligator mississippiensis*	Reptile
XP_061458755.1	- *Rhineura floridana*	Reptile
XP_054852824.1	- *Eublepharis macularius*	Reptile
XP_060105757.1	- *Heteronotia binoei*	Reptile
XP_056715197.1	- *Euleptes europaea*	Reptile
XP_029768487.1	- *Terrapene carolina triunguis*	Reptile
XP_034647404.1	- *Trachemys scripta elegans*	Reptile
XP_039361650.1	- *Mauremys reevesii*	Reptile
XP_053862991.1	- *Malaclemys terrapin pileata*	Reptile
XP_005298588.1	- *Chrysemys picta bellii*	Reptile
XP_032661302.1	- *Chelonoidis abingdonii*	Reptile
XP_050780243.1	- *Gopherus flavomarginatus*	Reptile
XP_028559681.1	- *Podarcis muralis*	Reptile
XP_030439197.1	- *Gopherus evgoodei*	Reptile

XP_053218286.1	- *Podarcis raffonei*	Reptile
CAI5789519.1	- *Podarcis lilfordi*	Reptile
XP_034990737.1	- *Zootoca vivipara*	Reptile
XP_033025471.1	- *Lacerta agilis*	Reptile
BAK64200.1	- *Elaphe quadrivirgata*	Reptile
BAK64199.1	- *Takydromus tachydromoides*	Reptile
XP_044296362.1	- *Varanus komodoensis*	Reptile
XP_015268723.1	- *Gekko japonicus*	Reptile
XP_015671282.1	- *Protobothrops mucrosquamatus*	Reptile
XP_053558084.1	- *Bombina bombina*	Amphibian
XP_053325177.1	- *Spea bombifrons*	Amphibian
XP_056384838.1	- *Hyla sarda*	Amphibian
XP_044144684.1	- *Bufo gargarizans*	Amphibian
XP_040207591.1	- *Rana temporaria*	Amphibian
XP_040272519.1	- *Bufo bufo*	Amphibian
CAH2295171.1	- *Pelobates cultripes*	Amphibian
XP_002932129.1	- *Xenopus tropicalis*	Amphibian
XP_018116507.1	- *Xenopus laevis*	Amphibian
XP_029427154.1	- *Rhinatrema bivittatum*	Amphibian
NP_001036195.1	- *Danio rerio*	Fish
XP_056313495.1	- *Danio aesculapii*	Fish
XP_048044578.1	- *Megalobrama amblycephala*	Fish
XP_056119822.1	- *Rhinichthys klamathensis*	Fish
XP_059404592.1	- *Carassius carassius*	Fish
XP_057176097.1	- *Triplophysa rosa*	Fish
RXN30848.1	- *Labeo rohita*	Fish
KAA0716739.1	- *Triplophysa tibetana*	Fish
XP_056603065.1	- *Triplophysa dalaica*	Fish
XP_055054651.1	- *Misgurnus anguillicaudatus*	Fish
XP_017564796.2	- *Pygocentrus nattereri*	Fish
XP_030646877.1	- *Chanos chanos*	Fish
XP_036455128.1	- *Colossoma macropomum*	Fish
XP_049326215.1	- *Astyanax mexicanus*	Fish
XP_029915996.1	- *Myripristis murdjan*	Fish
XP_038868660.1	- *Salvelinus namaycush*	Fish
XP_055742245.1	- *Salvelinus fontinalis*	Fish
XP_023834885.1	- *Salvelinus alpinus*	Fish
XP_029618120.1	- *Salmo trutta*	Fish
XP_036383444.1	- *Megalops cyprinoides*	Fish
XP_042278445.1	- *Thunnus maccoyii*	Fish
XP_056131525.1	- *Lampris incognitus*	Fish
XP_041957986.1	- *Alosa sapidissima*	Fish
XP_020345137.1	- *Oncorhynchus kisutch*	Fish

XP_035591377.1	- *Oncorhynchus keta*	Fish
XP_036791455.1	- *Oncorhynchus mykiss*	Fish
XP_024295139.1	- *Oncorhynchus tshawytscha*	Fish
XP_005999492.1	- *Latimeria chalumnae*	Fish
XP_056888499.1	- *Takifugu flavidus*	Fish
XP_042344673.1	- *Plectropomus leopardus*	Fish

Supplementary table 2:

Amino acid no	Cytosol	PM	ER	vacuolar	vesicles of secretory system	Golgi	nuclear	Mito	peroxisome
1-871	--	39.1	43.5	--	4.3	4.3	4.3	4.3	--
61-871	--	52.2	43.5	--	--	--	4.3	--	--
121-871	--	47.8	43.5	--	--	--	4.3	4.3	--
181-871	--	34.8	43.5	4.3	4.3	4.3	4.3	4.3	--
241-871	--	43.5	39.1	--	4.3	4.3	4.3	4.3	--
301-871	--	56.5	26.1	--	--	4.3	4.3	8.7	--
361-871	--	60.9	30.4	--	4.3	--	--	4.3	--
421-871	--	56.5	30.4	--	4.3	4.3	--	4.3	--
481-871	--	22.2	33.3	22.2	11.1	--	--	11.1	--
541-871	--	--	66.7	11.1	--	11.1	--	11.1	--
601-871	--	--	66.7	--	--	11.1	--	11.1	--
661-871	39.1	--	13	--	4.3	4.3	17.4	17.4	4.3
721-871	56.5	--	--	--	--	--	21.7	13	--
781-871							69.6		
601-840	--	--	66.7	--	--	11.1	--	11.1	--
601-660	11.1		22.2			44.4		22.2	
601-630	13	--	26.1	4.3	--	21.7	8.7	26.1	--
590-630	21.7		17.4	4.3		13		43.5	
592-630	17.4	--	21.7	4.3	--	8.7	--	47.8	--

Table represents predicted score for subcellular organelles localization for full-length hTRPV4 and its systemic deleted sequences as determined by the WoLF PSORT II prediction software.

Supplementary table 3:

a.

iPSORT Prediction

Predicted as: *not having signal or mitochondrial targeting peptide*

Sequence (Type: nonplant)

```
  1 MADSS EGPRA GPGEV AELPG DESGT PGGEA FPLSS LANLF EGEDG SLSPS
 51 PADAS RPAGP GDGRP NLRMK FQGAF RKGVP NPIDL LESTL YESSV VPGPK
101 KAPMD SLFDY GTYRH HSSDN KRWRK KIIEK QPQSP KAPAP QPPPI LKVFN
151 RPILF DIVSR GSTAD LDGLL PFLLT HKKRL TDEEF REPST GKTCL PKALL
201 NLSNG RNDTI PVLLD IAERT GNMRE FINSP FRDIY YRGQT ALHIA IERRC
251 KHYVE LLVAQ GADVH AQARG RFFQP KDEGG YFYFG ELPLS LAACT NQPHI
301 VNYLT ENPHK KADMR RQDSR GNTVL HALVA IADNT RENTK FVTKM YDLLL
351 LKCAR LFPDS NLEAV LNNDG LSPLM MAAKT GKIGI FQHII RREVT DEDTR
401 HLSRK FKDWA YGPVY SSLYD LSSLD TCGEE ASVLE ILVYN SKIEN RHEML
451 AVEPI NELLR DKWRK FGAVS FYINV VSYLC AMVIF TLTAY YQPLE GTPPY
501 PYRTT VDYLR LAGEV ITLFT GVLFF FTNIK DLFMK KCPGV NSLFI DGSFQ
551 LLYFI YSVLV IVSAA LYLAG IEAYL AVMVF ALVLG WMNAL YFTRG LKLTG
601 TYSIM IQKIL FKDLF RFLLV YLLFM IGYAS ALVSL LNPCA NMKVC NEDQT
651 NCTVP TYPSC RDSET FSTFL LDLFK LTIGM GDLEM LSSTK YPVVF IILLV
701 TYIIL TFVLL LNMLI ALMGE TVGQV SKESK HIWKL QWATT ILDIE RSFPV
751 FLRKA FRSGE MVTVG KSSDG TPDRR WCFRV DEVNW SHWNQ NLGII NEDPG
801 KNETY QYYGF SHTVG RLRRD RWSSV VPRVV ELNKN SNPDE VVVPL DSMGN
851 PRCDG HQQGY PRKWR TDDAP L
```

Values used for reasoning

Node	Answer	View	Substring	Value(s)	Plot
1. Signal peptide?	No	Average Hydropathy (KYTJ820101)	[6,20]	-0.653333 (>= 0.953? No)	show
2. Mitochondrial ?	No	Average Net Charge (KLEP840101)	[1,30]	-0.2 (>= 0.083? No)	show
		Indexing: AI1<br>Pattern: 221121122 (ins/del <= 3)	[1,30]	MADSSEGPRAGPGEVAELPGDESGTPGGEA<br>220220021202002202200020220002<br>NOMATCH	--

* This color means "not used".

Name	Alphabet Indexing		
	0	1	2
AI1	DEGHKN	IR	ACFLMPQSTVWY
AI2	ACDEFGHLMNQSTVWY	KR	IP

Return to iPSORT Home

b.

iPSORT Prediction

Predicted as: *having a mitochondrial targeting peptide*

Sequence (Type: nonplant)

```
1 FTRGL KLTGT YSIMI QKILF KDLFR FLLVY LLFMI GYAS
```

Values used for reasoning

Node	Answer	View	Substring	Value(s)	Plot
1. Signal peptide?	No	Average Hydropathy (KYTJ820101)	[6,20]	0.706667 (>= 0.953? No)	show
2. Mitochondrial ?	Yes	Average Net Charge (KLEP840101)	[1,30]	0.133333 (>= 0.083? Yes)	show
		Indexing: AI1<br>Pattern: 221121122 (ins/del <= 3)	[1,30]	FTRGLKLTGTYS-IM-I-QKILFKDLFRFLLVY<br>22102022022-12-1-201220022122222<br>221121122	--

* This color means "not used".

Name	Alphabet Indexing		
	0	1	2
AI1	DEGHKN	IR	ACFLMPQSTVWY
AI2	ACDEFGHLMNQSTVWY	KR	IP

Return to iPSORT Home

Prediction of mitochondrial-target sequence by iPSORT software. a. Full length hTRPV4 (1-871 aa) sequence was predicted for probable mitochondria target sequence. It shows no target sequence is present. **b.** TRPV4-MTS sequence (592-630 aa) was used for prediction of mitochondrial target sequence. It shows the presence of a target sequence.

Supplementary table 4:

Sequence of hTRPV4	Length (aa)	mTP Predicted score	Colocalization with Mitochondria
hTRPV4	1-871	0.06	Yes
TM	466-711	0.05	No
MTS	592-630	0.12	Yes
	L596P	0.24	Yes
	R616Q	0.12	Yes
	F617L	0.14	Yes
	L618P	0.20	Yes
	V620I	0.11	Yes
V4-Nt	1-465	0.08	No
V4-Ct	718-871	0.22	Yes

Table represents mitochondrial predicted score of full length TRPV4, Transmembrane region (TM), Predicted mitochondrial target sequence (592-630aa), different naturally occurring point mutants at the MTS region, mitochondrial N terminal sequence and C terminal sequence by Deeplock 2.0 software.
